# Recruitment of emergency department patients to a prospective observational study

**DOI:** 10.1186/s12245-024-00717-y

**Published:** 2024-10-07

**Authors:** Claire Shubeck, Hans Reyes Garay, Bret P. Nelson, Marcee Wilder, Aleksandra Degtyar, Megan Lukas, Lauren Gordon, George T. Loo, Bernice Coleman, Lynne D. Richardson, Kimberly Souffront

**Affiliations:** 1 Department of Emergency Medicine, Icahn School of Medicine at Mount Sinai, 1 Gustave L. Levy Place, New York, NY 10029 USA; 2 Institute for Health Equity Research, Icahn School of Medicine at Mount Sinai, 1 Gustave L. Levy Place, New York, NY 10029 USA; 3 Center for Nursing Research and Innovation, Mount Sinai Health System, 1 Gustave L. Levy Place, New York, NY 10029 USA; 4 Department of Cardiology, Mount Sinai Morningside, 440 W 114th Street Suite 220, New York, NY 10025 USA; 5 Department of Population Health Science and Policy, Icahn School of Medicine at Mount Sinai, 1 Gustave L. Levy Place, New York, NY 10029 USA; 6 Cedars-Sinai Medical Center, 8711 3rd Street, TSB, Room 1132, Los Angeles, CA 90048 USA; 7 Department of Artificial Intelligence and Human Health, Icahn School of Medicine at Mount Sinai, 1468 Madison Ave 11th Floor, New York, NY 10029 USA; 8 Tufts University School of Medicine, 136 Harrison Avenue, Boston, MA 02111 USA

**Keywords:** Feasibility, Recruitment, Emergency research, Hypertension, Prospective observational, Asymptomatic hypertension, Racial and ethnic diversity, Diversity, Health equity

## Abstract

**Background:**

The dynamic environment of the emergency department (ED) poses unique challenges to the execution of well-designed research. There is limited investigation into the viability of studies conducted in the ED. This paper offers a systematic evaluation of our recruitment of emergency patients for a prospective observational research study, shedding light on the intricate landscape of research feasibility within the ED setting.

**Results:**

Research coordinators dedicated 2816.83 h to screening, recruiting, and enrolling patients between June 2018 and September 2023, having to stop recruitment twice due to financial constraints and the COVID-19 pandemic. 485 patients were approached and 84 of them were enrolled, resulting in a 31.94% enrollment rate, with approximately 2.8 participants recruited per month. Of those enrolled, 77 completed all study endpoints. Most participants were Hispanic (n = 44; 52.3%) and/or Black (n = 37; 44%), middle-aged (µ = 51.7 years), and female (n = 48; 57.1%). Participant recruitment was challenged by competing mindsets, the COVID-19 pandemic, and high staff turnover.

**Conclusions:**

Recruiting emergency patients for a prospective observational study is feasible given adequate staffing and financial resources. Standardizing feasibility assessments for the recruitment of patients in the emergency department is important to the success of future study.

## Introduction

Conducting clinical research in the emergency department (ED) presents unique challenges that are amplified by the fast-paced, high-pressure nature of emergency care, which differs from the more controlled setting of outpatient research [1–4]. Specifically, obtaining informed consent from acutely ill patients can be complicated by their cognitive state, and integrating research protocols into the ED’s fast-moving clinical workflow adds further complexity [5]. Addressing these ED-specific challenges is essential for achieving study goals and advancing emergency medicine research. The importance of racial and ethnic minority research participation is vital to the validity of any study but often requires additional strategies and resources [6, 7]. Patients who have limited English proficiency may be excluded if consent forms and study materials are not available in their native language, and they are less likely to enroll when research staff are not culturally and linguistically competent [8–10]. Culturally congruent study designs that minimize barriers to research participation is crucial to enhance the rigor of research [11].

Assessing the feasibility of conducting research in the ED is not well standardized. This paper describes our recruitment efforts for a prospective observational study on asymptomatic hypertension, highlighting the complexities encountered, and shedding light on the feasibility of recruiting participants in the ED setting.

## Organizational framework

We utilized Stewart et al.‘s [12] comprehensive eight-step organizational framework to systematically assess the feasibility of recruiting ED patients to a prospective observational study (Table 1). By adopting this framework, we streamlined our feasibility assessment process and enhanced the comprehensiveness of our study, ultimately contributing to the robustness and integrity of our research findings.

**Table 1:** Organizational framework for assessing recruitment feasibility

1.	Specify recruitment goals: target population, desired diversity, and subgroup sample sizes
2.	Specify recruitment process by stage
3.	Establish a tracking system for each individual: contact tracking form
4.	Establish a tracking database for monitoring processes and results
5.	Implement recruitment processes and monitor each individual’s progress
6.	Summarize recruitment results from the tracking database, including by targeted subgroups: real time and final results
7.	Calculate and interpret measures of feasibility, including by targeted subgroups: were goals met
8.	If goals not met, utilize tracking data to modify methods for a larger study

Reproduced with permission from Stewart et al. [12]

## Methods

### Study design

In this descriptive study, we conducted a manual review of our recruitment and retention data for an observational study once that study was concluded. The parent study sought to determine the utility of a blood biomarker as a proxy for detecting subclinical heart disease in emergency patients with asymptomatic hypertension and how nurse-led risk communication impacts blood pressure control at 6 months [13, 14]. We present our recruitment feasibility data in eight specific steps as outlined by Stewart et al. [12].

### Stewart et al. framework for assessing recruitment feasibility

#### Step 1: Specify recruitment goals – target population, desired diversity, and subgroup sample sizes

The parent study began after approval from the institutional review board (IRB) (#18–00197) at the Icahn School of Medicine at Mount Sinai. We included adult (≥ 18 years) patients whose initial blood pressure readings met or exceeded 160/100 mmHg and whose second readings were at or above 140/90 mmHg, provided they were pending discharge and were proficient in English or Spanish. We excluded patients with a diagnosis of congestive heart failure, renal insufficiency, and atrial fibrillation or who were pregnant, incarcerated, cognitively unable to provide informed consent, or experiencing symptoms of hypertension, such as chest pain, paresthesia, or shortness of breath as identified by triage or provider notes and confirmed with the patient before data collection [13, 14].

No one was excluded from participation based on sex/gender, race, or ethnicity. Given the ethnic and racially diverse population presenting to the EDs located in New York City, NY, which are approximately 35% Hispanic, 29% African American, 19% White, 1% Asian, and 16% other, we aimed to recruit a diverse sample based on race/ethnicity, sex/gender, and language.

A sample size calculation was conducted a priori. Assuming a 95% confidence interval and a precision of 0.05 we determined that *n* = 76 was necessary to achieve a 97% sensitivity and 90% specificity of a blood biomarker for detecting subclinical heart disease in emergency patients with asymptomatic stage 2 hypertension (Table 2). The targeted planned distribution of subjects by sex, race, and ethnicity for the proposed research was also conducted a priori. Based on the racial and ethnic demographics of the population presenting in the ED at our hospital, we projected an enrollment of 13 individuals from more than one race and/or ethnicity, 22 African Americans, 26 Hispanics, 14 Caucasians, and 1 Asian, for a total of 76 participants.

**Table 2:** Sample size calculation

				Prevalence Rates (%)		
	Sensitivity	Specificity	Precision	Low	Medium	High
*p* = 0.05; CI^a^ = 95%	97%	90%		40.0	60.0	90.0
				Sample Size		
			0.01	2796	1864	1243
			0.015	1243	829	553
			0.025	448	299	199
			0.05	113	76	51
			0.07	58	39	26
			0.10	31	21	14

^a^CI = confidence interval

#### Step 2: Specify recruitment processes by stages

After IRB approval from the Icahn School of Medicine at Mount Sinai New York, a robust training program was designed for all personnel involved in the study, including program management, research coordinators, physicians, and sonographers. This role-specific training regimen spanned 80 h for each research team member and included practical elements, such as shadowing experienced personnel, to ensure strict adherence to the study protocol. For sonographers who performed an echocardiogram on the participants, any discordance in measurements during recruitment was promptly addressed through retraining and reeducation efforts by the lead physician sonographer (BN) either electronically or in person, guaranteeing the uniformity and accuracy of data collection. Six sonographers were trained to conduct the study protocol. For research coordinators, instruction covered a spectrum of skills, including training with the ED nurse educator on how to conduct an electrocardiogram (ECG) and venipuncture, which required 10 successful ECG and 10 venipuncture performances. Coordinators were also trained to take a patient’s blood pressure if nurses had not already recorded a second blood pressure measurement in the electronic health record, Epic [15, 16]. Eleven research coordinators were trained over the study period.

We recognized the linguistic diversity within our patient population and appointed a research coordinator specifically for patients who were only proficient in Spanish. We used a Spanish consent form to facilitate effective communication and ensure clear understanding for patients who were only proficient speaking and understanding in Spanish. Five (45%) research coordinators were Spanish speaking. Research coordinators self-identified as Black and/or Hispanic (*n* = 7; 64%); White (*n* = 3; 27%), and Asian (*n* = 1; 9%).

Research coordinators used Microsoft Teams to notify the sonographer at the beginning of each data collection day so that they were prepared to perform an echocardiogram on any new participants. Research coordinators used the Epic chat feature to inform the ED physician about prospective participants. Informed consent was obtained electronically, and data were recorded using the secure and HIPAA-compliant Research Electronic Data Capture (REDCap) system [17]. Patients received up to $100.00 compensation. A more detailed data collection procedure is detailed elsewhere [13, 14].

#### Step 3: Establish a tracking system for each individual – contact tracking form

Study eligibility was limited to ED patients who were treated at one of two ED sites within our eight-hospital healthcare system in New York City, NY; no external recruitment efforts were made. Each enrolled patient was assigned a distinct, password-protected record ID within REDCap, where our research coordinators recorded consent, endpoints, and patient information. This system allowed us to monitor each patient’s journey through the study, starting from the moment of consent.

#### Step 4: Establish tracking database for monitoring processes and results

Our clinical research coordinators served as the principal recruiters, conducting patient screening and enrollment for three 8-hour daytime shifts each week at one of two study sites. We harnessed Epic for data collection across both ED sites. At the end of each shift, research coordinators would record in a spreadsheet the number of patients screened, excluded, approached, enrolled, and completed. Coordinators also noted how many patients declined to participate, and in most cases, documented the reason for not enrolling. REDCap was used to oversee data for enrolled participants only whereas the spreadsheet was used as an overview for screening through enrollment.

#### Step 5: Implement recruitment processes and monitor each individual’s progress

Throughout this process, any challenges or issues arising during this step were promptly identified and resolved through close communication within our research team. Weekly meetings served as a forum for discussion, allowing our program manager to oversee recruitment data entry and report directly to the principal investigator.

## Results

### ***Step 6: Summarize recruitment results from the tracking database including by targeted subgroups – real time and final results***

Research coordinators dedicated 2816.83 h to screening, recruiting, and enrolling patients between June 2018 and September 2023.

**Fig. 1:**
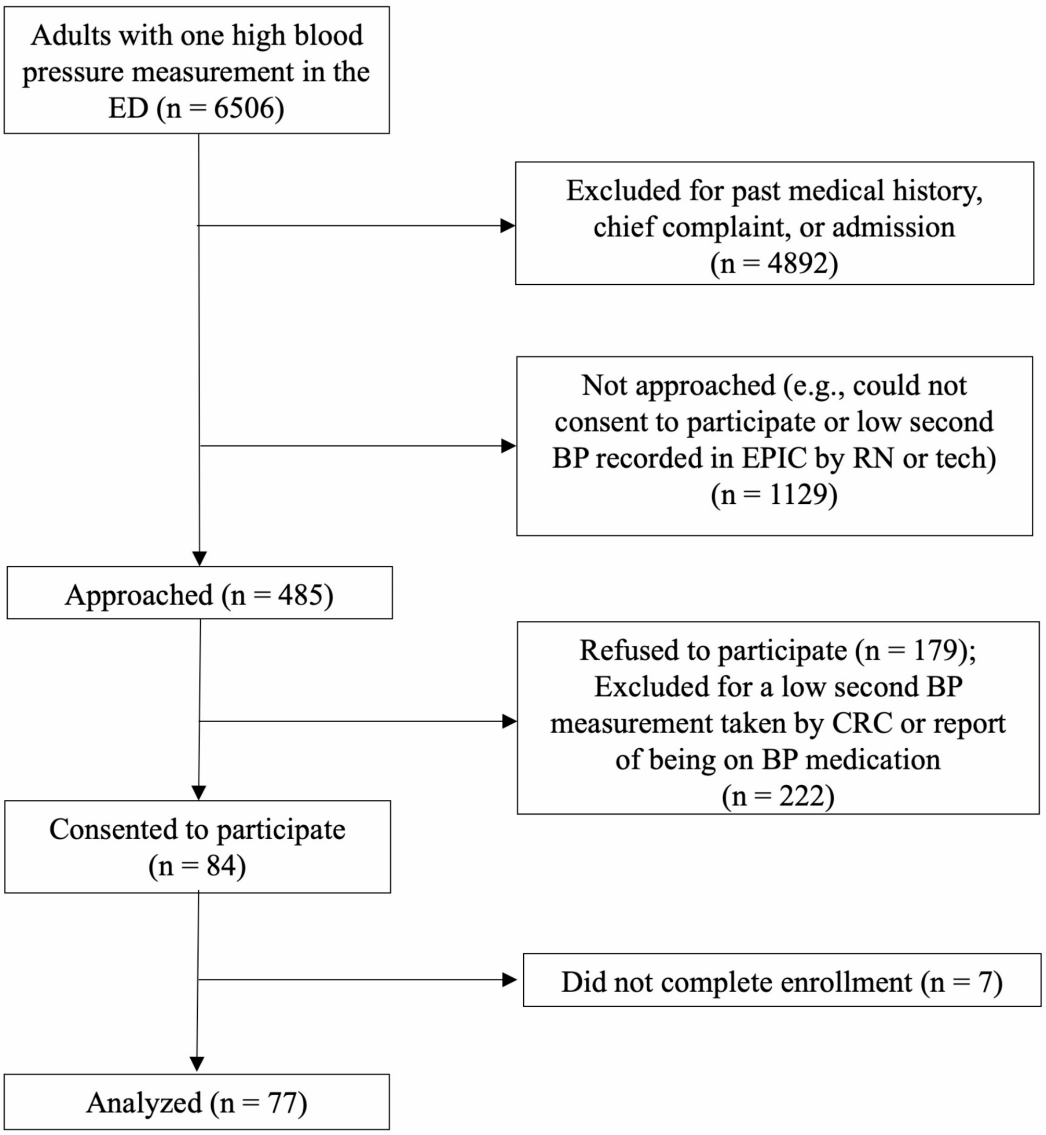
Flowchart of screening, recruitment, and enrollment of research participants ED = emergency department, BP = blood pressure, CRC = clinical research coordinator

We screened 6506 patients across two ED sites with an initial blood pressure reading ≥ 160/100 mmHg (Fig. 1). Excluding 6021 based on medical history, chief complaint, admission, a normal second blood pressure reading in Epic, or inability to provide informed consent (language barriers or cognitive impairment), we approached 485 patients. Among them, 179 refused and 222 were excluded due to a normal second blood pressure or reporting antihypertensive medication. Ultimately, 84 patients consented (31.94% enrollment rate); 77 completed all study endpoints (EKG, lab test, and sonogram), while 7 were missing at least one test. Approximately 16 participants were approached, and 2.8 participants were enrolled per month. We exceeded our target sample size by *n* = 1 as our recruitment shift for the day of the final enrollment was already set.

Race and ethnicity were collected during enrollment but not the screening process. Most participants enrolled were Hispanic (*n* = 44; 52.3%) and/or Black (*n* = 37; 44%), middle-aged (µ = 51.7 years), and female (*n* = 48; 57.1%). The mean initial systolic BP (sBP) was 171.89 mmHg (*n* = 84 (100%); SD = 14.35 mmHg) and diastolic BP (dBP) 100.26 mmHg (*n* = 84 (100%); SD = 13.28 mmHg). The mean second sBP was 162.35 mmHg (*n* = 74 (88%); SD = 18.42 mmHg) and dBP 94.93 mmHg (*n* = 74 (88%); SD = 15.15 mmHg).

**Fig. 2:**
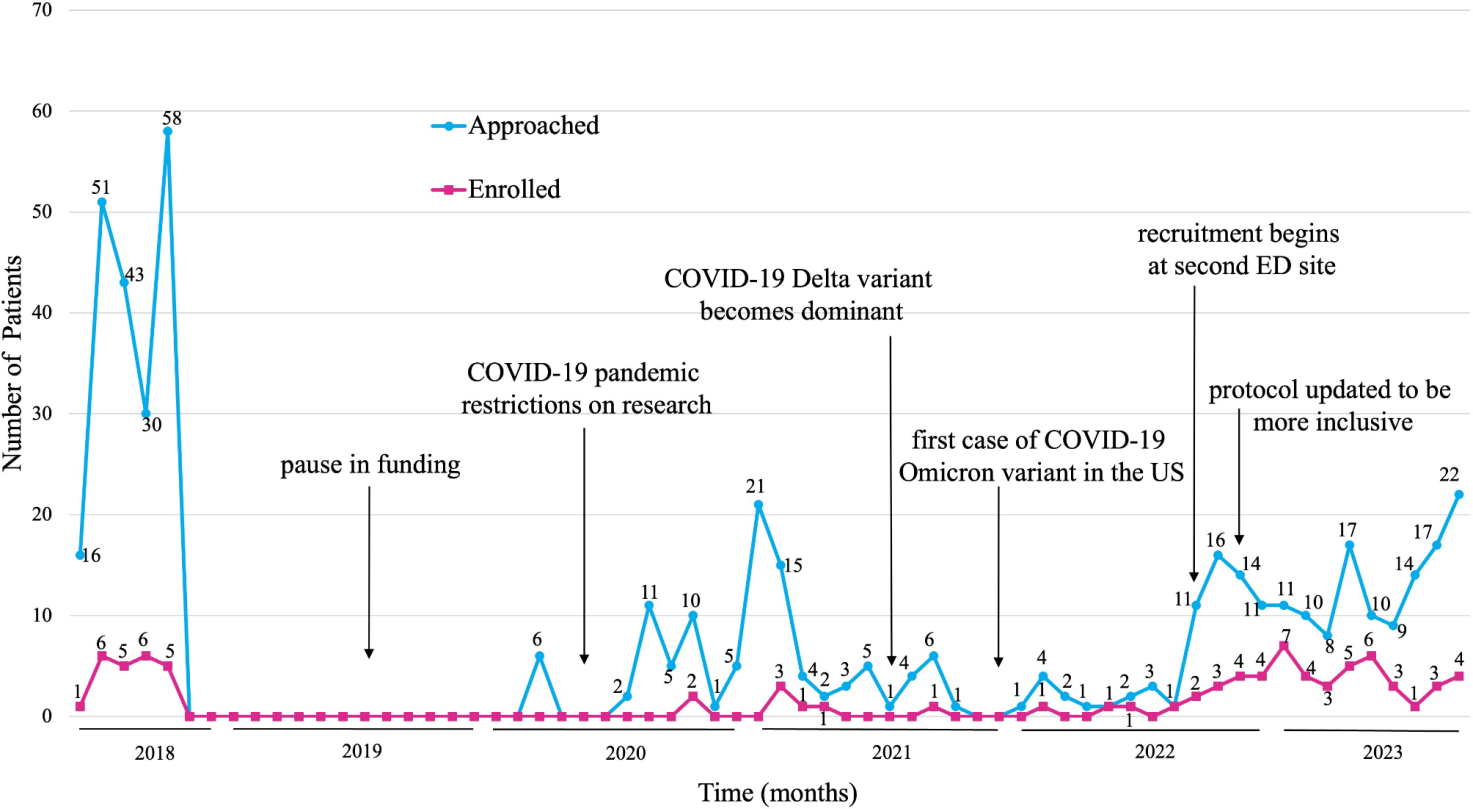
Number of patients approached and enrolled during the recruitment period

Our study encountered various barriers and facilitators that influenced the recruitment process described below.

#### Barriers to recruitment

1. *Competing Mindsets*: Patients in the ED are primarily focused on addressing their immediate health issues which can hinder their willingness to engage in research. Seven participants did not complete all three tests (EKG, lab test, or sonogram), primarily because of time constraints.
2. *COVID-19 Pandemic and Funding*: The impacts of several events described here are portrayed in Fig. 2. The onset of the COVID-19 pandemic disrupted the recruitment process and necessitated protocol changes to ensure we achieved our desired sample of *n* = 76. A temporary interruption in funding also slowed down recruitment, causing the study to appear stagnant in 2019. We then added another recruitment site within our healthcare organization which entailed reconfiguring logistics pertaining to study startup, gaining leadership support, and staff (re)training. We also addressed limited resources for conducting ultrasounds at the second site by collaborating with the cardiology department to perform them. However, this approach necessitated escorting patients out of the ED after discharge to a different area for the ultrasound, impacting the study’s efficiency and patient experience. Despite these hurdles, recruiting at this additional site boosted enrollment by increasing the pool of eligible patients.
3. *Staff Turnover*: High turnover among research coordinators, including those pursuing further education or transitioning to other careers, posed a recurring challenge. Over the course of the study, 11 research coordinators were involved. Each new addition required orientation and shadowing to ensure that the team was well-prepared to conduct all study-related tasks and that consistency in protocols were maintained. High turnover among sonographers added further complications. One career sonographer staffed by our ED conducted most sonograms because sonogram fellows had frequent conflicts with study-related meetings and tasks. The limited availability of a sonographer slowed down data collection.

#### Facilitators to recruitment

1. *Leadership support*: The research responsibilities of nursing staff needed to be balanced with their clinical workload. Effective communication and regular meetings with nursing staff and faculty within the ED was vital for ensuring ED leadership support for research objectives and smooth recruitment. The leadership support our study experienced may be more likely in research-intense health systems like ours and less so in other ED settings.
2. *Incentives*: Offering incentives to participants, such as compensation for their time and involvement, proved effective in encouraging participation.
3. *Spanish-proficient coordinators*: Spanish-speaking research coordinators facilitated effective communication and broadened our reach. However, high staff turnover limited the availability of Spanish-speaking research coordinators which meant, occasionally, patients who met inclusion criteria but spoke Spanish were not able to be approached.

### ***Step 7: Calculate and interpret measures of feasibility***,*** including by targeted subgroups – were goals met?***

Our goals were satisfactorily met. Our objectives were centered on specific metrics, most notably the number of participants successfully enrolled in the study. With concerted effort and meticulous recruitment strategies, we successfully achieved our desired sample size (*n* = 77 having all three tests), signifying the successful execution of our research plan. In our pursuit of diversity within our study, we were equally successful. Our sample demonstrated a commendable level of diversity across various demographic factors, including race, ethnicity, and sex/gender. This accomplishment reinforces the comprehensive and inclusive nature of our study.

We encountered a minimal issue wherein seven participants had to be replaced due to missing data (EKG, lab test, or sonogram). Nevertheless, our stringent data management and systematic approach to data collection allowed for swift resolution, ensuring the continued integrity of our study.

## Discussion

### ***Step 8: If goals not met***,*** utilize tracking data to modify methods for a larger study***

Our study demonstrated that it is feasible to recruit ED patients to research in the context of a condition frequently encountered in ED settings, elevated blood pressure. Although we achieved our recruitment goals, we experienced a slow pace of 2.8 participants enrolled per month. Other studies with hypertensive patients in the ED have reported higher enrollment rates than ours [18–20], but it is likely that the COVID-19 pandemic’s impact on the ED and research in this context is partially responsible for this difference. Institutional restrictions on research and a decrease in ED visits reduced opportunities for recruitment and prolonged the recruitment period. Our recruitment trajectory shows the gradual increase in recruitment after limitations on research were lifted.

Our enrollment rate is lower than that of clinical research studies in primary care and outpatient settings where a majority of patients who are approached agree to participate [21–23]. This difference in recruitment between settings reflects the unique set of challenges to conducting research in the ED. Researchers often face logistical difficulties in obtaining informed consent from patients, given the urgency of many ED cases and the inherent challenges in maintaining a confidential and supportive research environment [1]. Moreover, the ED frequently serves historically marginalized and underserved populations, such as those without access to regular healthcare which raises ethical and logistical concerns related to informed consent, confidentiality, and the need for research that is representative of all demographics [24]. Addressing these issues is pivotal for advancing clinical knowledge and improving healthcare outcomes for all patients, regardless of their racial and ethnic background or clinical condition.

We prepared for ED-specific obstacles to research and adjusted mid-study as needed. For example, in November 2022, we stopped excluding patients who had taken blood pressure medication within the last three months. With this decision, we aimed to make our study inclusive of those who might have untreated conditions or might need intervention. This adjustment expanded the pool of eligible patients and broadened our assessment of the study population.

We addressed language barriers by employing research coordinators who were proficient speaking Spanish, but they were inconsistently available. The varied schedule of the sonographers was also challenging to align with that of the coordinators. Based on our assessment, the most impactful adjustments to recruitment in the same context would be to increase the availability of sonographers and research coordinators who were proficient speaking Spanish to address language barriers and patients’ negative perception of required time and effort to enroll.

Employing Stewart et al.‘s structured organizational framework ensured a methodical and robust approach to recruitment, while our commitment to ethical practices maintained the integrity of our data. Furthermore, this analysis reveals the great extent of resources required to account for the challenges particular to research in the ED setting. As the parent study demonstrated, results from research performed in the ED are important because they can inform interventions for ED patients with chronic conditions. However, high levels of institutional support and financial resources are needed to perform this research. This is not reflected in the current attention granted by healthcare institutions and research centers to ED research; funding is significantly less for emergency research as compared to other specialties [25, 26]. Our analysis emphasizes the importance of growing the effort and support for rigorous and impactful emergency medicine research.

### Strengths and limitations

The specific setting of our study in two urban academic EDs may limit the generalizability of our findings. Our study also experienced a relatively low enrollment rate, with 31.94% of eligible patients approached agreeing to participate. This raises questions about the representativeness of our sample, as those who declined participation may differ from those who enrolled. Our study encountered missing data for seven participants which required their replacement. Understanding the patient population and the challenges of obtaining complete data in this setting is crucial.

Despite these limitations, our research demonstrated the feasibility of recruiting patients for studies in this challenging environment, and we successfully enrolled individuals reflecting the ED’s demographic diversity.

### Implications for future emergency practice

The findings of our study have several important implications for emergency care practice. Firstly, by shedding light on the feasibility of recruiting ED patients to prospective studies, we offer a pathway for the development of more effective interventions within the ED setting. Our success in enrolling a racially and ethnically diverse further underscores the importance of inclusive research practices in emergency care. Additionally, the utilization of a structured organizational framework for feasibility assessment, as demonstrated in our study, provides a valuable resource for emergency care practitioners to streamline and enhance their recruitment processes.

## Conclusions

This paper describes a comprehensive organizational framework to assess the feasibility of recruiting participants for research in the challenging ED setting. Through rigorous training and dedication to high-quality research, we overcame various obstacles, including the unique demands of the ED environment, the impact of the COVID-19 pandemic, recruitment logistics, staff turnover, and missing data. Leadership support, incentives, and research coordinators who were proficient speaking Spanish acted as important facilitators in our recruitment efforts. We successfully recruited a racially and ethnically diverse sample of patients, ultimately achieving our target sample size. Our findings not only contribute to understanding asymptomatic hypertension in the ED but also provide insights and tools for enhancing recruitment processes in emergency care research. Specifically, this feasibility assessment can inform future studies. We hope that our experiences can guide future research endeavors, foster the inclusion of historically underrepresented populations in clinical studies, and advance the quality of healthcare for all patients.

## Data Availability

No datasets were generated or analysed during the current study.

## Declarations

### Authors’ information

Dr. Kimberly Souffront is Principal Investigator. She is Associate Professor of Emergency Medicine and Health Equity Research at the Icahn School of Medicine at Mount Sinai, and Associate Director of the Center for Nursing Research and Innovation at the Mount Sinai Health System.

## References

[1] Shea S, Thomas Bigger J, Campion J, Fleiss JL, Rolnitzky LM, Schron E et al. Enrollment in clinical trials: Institutional factors affecting enrollment in the Cardiac Arrhythmia Suppression Trial (CAST). Controlled Clinical Trials [Internet]. 1992 Dec [cited 2023 Nov 15];13(6):466–86. https://linkinghub.elsevier.com/retrieve/pii/019724569290204D 10.1016/0197-2456(92)90204-d 1334819

[2] Bjornson-Benson WM, Stibolt TB, Manske KA, Zavela KJ, Youtsey DJ, Sonia Buist A. Monitoring recruitment effectiveness and cost in a clinical trial. Controlled Clinical Trials [Internet]. 1993 Apr [cited 2023 Nov 15];14(2):52–67. https://linkinghub.elsevier.com/retrieve/pii/0197245693900248 10.1016/0197-2456(93)90024-8 8500313

[3] Durkin DA, Kjelsberg MO, Sonia Buist A, Connett JE, Owens GR. Recruitment of participants in the Lung Health Study, I: Description of Methods. Controlled Clinical Trials [Internet]. 1993 Apr [cited 2023 Nov 15];14(2):20–37. https://linkinghub.elsevier.com/retrieve/pii/0197245693900226 10.1016/0197-2456(93)90022-6 8500310

[4] Williams GH. Funding for Patient-Oriented Research: Critical Strain on a Fundamental Linchpin. JAMA [Internet]. 1997 Jul 16 [cited 2023 Nov 22];278(3):227. http://jama.jamanetwork.com/article.aspx?doi=10.1001/jama.1997.03550030067036 9218670

[5] Cofield SS, Conwit R, Barsan W, Quinn J. Recruitment and Retention of Patients into Emergency Medicine Clinical Trials. Academic Emergency Medicine [Internet]. 2010 Oct [cited 2023 Nov 8];17(10):1104–12. https://onlinelibrary.wiley.com/doi/10.1111/j.1553-2712.2010.00866.x 10.1111/j.1553-2712.2010.00866.x PMC3058592 21040112

[6] George S, Duran N, Norris K. A Systematic Review of Barriers and Facilitators to Minority Research Participation Among African Americans, Latinos, Asian Americans, and Pacific Islanders. Am J Public Health [Internet]. 2014 Feb [cited 2023 Nov 8];104(2):e16–31. 10.2105/AJPH.2013.301706 10.2105/AJPH.2013.301706 PMC3935672 24328648

[7] Oh SS, Galanter J, Thakur N, Pino-Yanes M, Barcelo NE, White MJ et al. Diversity in Clinical and Biomedical Research: A Promise Yet to Be Fulfilled. PLoS Med [Internet]. 2015 Dec 15 [cited 2023 Nov 8];12(12):e1001918. 10.1371/journal.pmed.1001918 10.1371/journal.pmed.1001918 PMC4679830 26671224

[8] Wendler D, Kington R, Madans J, Wye GV, Christ-Schmidt H, Pratt LA et al. Are Racial and Ethnic Minorities Less Willing to Participate in Health Research? Gill P, editor. PLoS Med [Internet]. 2005 Dec 6 [cited 2023 Nov 22];3(2):e19. 10.1371/journal.pmed.0030019 10.1371/journal.pmed.0030019 PMC1298944 16318411

[9] Durant RW, Wenzel JA, Scarinci IC, Paterniti DA, Fouad MN, Hurd TC, et al. Perspectives on barriers and facilitators to minority recruitment for clinical trials among cancer center leaders, investigators, research staff, and referring clinicians: enhancing minority participation in clinical trials (EMPaCT). Cancer. 2014;120(0 7):1097–105. 10.1002/cncr.28574 10.1002/cncr.28574 PMC4395557 24643647

[10] Barrett NJ, Ingraham KL, Hawkins TV, Moorman PG. Engaging African Americans in Research: The Recruiter’s Perspective. Ethn Dis [Internet]. 2017 Dec 7 [cited 2023 Nov 22];27(4):453. https://www.ethndis.org/edonline/index.php/ethndis/article/view/753 10.18865/ed.27.4.453 PMC5720956 29225447

[11] Razon N, Hessler D, Bibbins-Domingo K, Gottlieb L. How Hypertension Guidelines Address Social Determinants of Health: A Systematic Scoping Review. Medical Care [Internet]. 2021 Dec [cited 2023 Nov 22];59(12):1122–9. https://journals.lww.com/10.1097/MLR.0000000000001649 10.1097/MLR.0000000000001649 PMC8597925 34779795

[12] Stewart AL, Nápoles AM, Piawah S, Santoyo-Olsson J, Teresi JA. Guidelines for evaluating the feasibility of recruitment in Pilot studies of diverse populations: an overlooked but important component. Ethn Dis. 2020;30(Suppl 2):745–54. 10.18865/ed.30.S2.745 10.18865/ed.30.S2.745 PMC7683033 33250621

[13] Souffront K, Nelson B, Murphy M, Garay HR, Gordon L, Matos T et al. Stage B Heart Failure Is Ubiquitous in Emergency Patients with Asymptomatic Hypertension. Western Journal of Emergency Medicine: Integrating Emergency Care with Population Health [Internet]. 2024; https://escholarship.org/uc/item/0t02p5ch 10.5811/westjem.17990 PMC11000548 38596912

[14] Souffront K, Shubeck C, Nelson BP, Lukas M, Gordon L, Garay HR et al. Brief risk communication for sustained asymptomatic hypertension in the emergency department. J Emerg Nurs. [accepted August 13th, 2024]. Manuscript ID JEN-D-24-00176 10.1016/j.jen.2024.08.001 39320299

[15] EPIC Research. https://epicresearch.org/submission-guidelinesz. Accessed 8 May 2023.

[16] Levy P, Ye H, Compton S, Zalenski R, Byrnes T, Flack JM, et al. Subclinical hypertensive heart disease in black patients with elevated blood pressure in an inner-city emergency department. Ann Emerg Med. 2012;60(4):467–e4741. 10.1016/j.annemergmed.2012.03.030 10.1016/j.annemergmed.2012.03.030 22658278

[17] Patridge EF, Bardyn TP. Research Electronic Data Capture (REDCap). jmla [Internet]. 2018 Jan 12 [cited 2023 Oct 23];106(1). http://jmla.pitt.edu/ojs/jmla/article/view/319

[18] Goldberg EM, Wilson T, Jambhekar B, Marks SJ, Boyajian M, Merchant RC. Emergency Department-provided home blood pressure devices can help detect undiagnosed hypertension. High Blood Press Cardiovasc Prev. 2019;26(1):45–53. 10.1007/s40292-019-00300-0 10.1007/s40292-019-00300-0 PMC6407636 30659517

[19] Meurer WJ, Dome M, Brown D, Delemos D, Oska S, Gorom V et al. Feasibility of Emergency Department–initiated, Mobile Health Blood Pressure Intervention: An Exploratory, Randomized Clinical Trial. Smith SW, editor. Academic Emergency Medicine [Internet]. 2019 May [cited 2023 Dec 6];26(5):517–27. https://onlinelibrary.wiley.com/doi/10.1111/acem.13691 10.1111/acem.13691 PMC6945785 30659702

[20] Skolarus LE, Dinh M, Kidwell KM, Lin CC, Buis LR, Brown DL et al. Reach Out Emergency Department: A Randomized Factorial Trial to Determine the Optimal Mobile Health Components to Reduce Blood Pressure. Circ: Cardiovascular Quality and Outcomes [Internet]. 2023 May [cited 2023 Dec 6];16(5). https://www.ahajournals.org/doi/10.1161/CIRCOUTCOMES.122.009606 10.1161/CIRCOUTCOMES.122.009606 37192282

[21] Bennett GG, Steinberg D, Askew S, Levine E, Foley P, Batch BC, et al. Effectiveness of an app and provider counseling for obesity treatment in primary care. Am J Prev Med. 2018;55(6):777–86. 10.1016/j.amepre.2018.07.005 10.1016/j.amepre.2018.07.005 PMC6388618 30361140

[22] Lopes S, Mesquita-Bastos J, Garcia C, Bertoquini S, Ribau V, Teixeira M, et al. Effect of Exercise Training on ambulatory blood pressure among patients with resistant hypertension: a Randomized Clinical Trial. JAMA Cardiol. 2021;6(11):1317–23. 10.1001/jamacardio.2021.2735 10.1001/jamacardio.2021.2735 PMC8340008 34347008

[23] Donini LM, Cuzzolaro M, Gnessi L, Lubrano C, Migliaccio S, Aversa A, et al. Obesity treatment: results after 4 years of a Nutritional and Psycho-Physical Rehabilitation Program in an outpatient setting. Eat Weight Disord. 2014;19(2):249–60. 10.1007/s40519-014-0107-6 10.1007/s40519-014-0107-6 24577668

[24] Burchard EG, Oh SS, Foreman MG, Celedón JC. Moving toward *True* Inclusion of Racial/Ethnic Minorities in Federally Funded Studies. A Key Step for Achieving Respiratory Health Equality in the United States. Am J Respir Crit Care Med [Internet]. 2015 Mar 1 [cited 2023 Nov 15];191(5):514–21. https://www.atsjournals.org/doi/10.1164/rccm.201410-1944PP 10.1164/rccm.201410-1944PP PMC4384771 25584658

[25] Wright SW, Wrenn K. Funding in the emergency medicine literature: 1985 to 1992. Ann Emerg Med. 1994;23(5):1077–81. 10.1016/s0196-0644(94)70107-5 10.1016/s0196-0644(94)70107-5 8185103

[26] Singer AJ, Homan CS, Stark MJ, Werblud MC Jr., Hollander HCT. Comparison of types of Research Articles published in Emergency Medicine and Non-emergency Medicine journals. Acad Emerg Med. 1997;4(12):1153–8. 10.1111/j.1553-2712.1997.tb03699.x 10.1111/j.1553-2712.1997.tb03699.x 9408432

